# An environmental scan of residential treatment service provision in Ontario

**DOI:** 10.1186/s13011-023-00586-3

**Published:** 2023-12-12

**Authors:** Farihah Ali, Justine Law, Cayley Russell, Nikki Bozinoff, Brian Rush

**Affiliations:** 1 Institute for Mental Health Policy Research, Centre for Addiction and Mental Health (CAMH), 33 Ursula Franklin St, Toronto, Ontario M5S 2S1 Canada; 2 Ontario Node, Canadian Research Initiative in Substance Misuse (CRISM), Centre for Addiction and Mental Health (CAMH), 33 Ursula Franklin St, Toronto, Ontario M5S 2S1 Canada; 3 Campbell Family Mental Health Research Institute, Centre for Addiction and Mental Health, Toronto, Ontario Canada; 4 Addictions Division, Centre for Addiction and Mental Health, Toronto, Ontario Canada; 5 Department of Family and Community Medicine, University of Toronto, Toronto, Ontario Canada

**Keywords:** Substance use disorders, Residential treatment, Opioid agonist treatment, Ontario, Canada

## Abstract

**Background:**

Ontario has one of the highest rates of substance-related harms in Canada. Residential treatment programs in the province provide a variety of in-house treatment services to support the needs of individuals with substance use disorders (SUD). However, these programs are not standardized, often varying in the type, quality, and availability of services offered, including evidence-based interventions such as Opioid Agonist Treatment (OAT). Local treatment systems are also rather fragmented and complex to navigate, creating barriers for potential services users to identify and make informed choices on available treatment options.

**Methods:**

Between May to August 2023, we conducted an environmental scan to capture available information on all publicly-funded residential treatment programs in Ontario using the ConnexOntario service portal, a government-funded, health services information platform. Data were captured on organization name, geographical location, program description, program type (residential addictions treatment or supportive recovery programs), eligibility criteria, target population, the program’s OAT policies, number of available beds, minimum and maximum length of stay, projected wait times, funding source, and associated fees for program admission. Data were extracted and organized by geographic region, and findings were presented descriptively.

**Results:**

A total of 102 residential addiction treatment programs and 36 residential supportive recovery programs in Ontario were identified. The scan noted substantial regional variations in program availability and wait times, along with a lack of programs tailored to unique populations such as women, youth, and Indigenous peoples. There is also a paucity of publicly-available information on program offerings, including detailed specifics on OAT policies within residential treatment programs that are crucial to ensuring that the services being offered are safe and grounded in evidence-based practice.

**Conclusions:**

Findings from the scan highlight notable gaps in program types, offerings, and availability among residential treatment programs in the province, including a lack of standardization on OAT policies across programs. Efforts should be made to ensure access to treatment-specific program information relevant to potential service users and to enhance coordinated access to residential treatment services in the province.

**Supplementary Information:**

The online version contains supplementary material available at 10.1186/s13011-023-00586-3.

## Background

Within Canada, Ontario is among the provinces with the highest rates of substance-related harms. There were a total of 2543 opioid-toxicity deaths (a rate of 16.8/100,000 population) and 2023 opioid-related poisoning hospitalizations (a rate of 13.4/100,000 population) in Ontario in 2022 [1, 2], as well as the highest number of stimulant-related hospitalizations amongst the provinces that year, at 844 [1]. Moreover, rising trends of polysubstance use, driven by an increasingly contaminated drug supply which now predominantly includes fentanyl and non-prescription benzodiazepines, is contributing to the increase in drug-toxicity hospitalizations and deaths observed in the province [3, 4]. This reality further complicates overdose responses and signals a need for an expansion in access to supports and services for the treatment and management of substance use disorders (SUD) [2].

The most recent data on treatment access in Ontario indicates that in 2017-2018, one in 217 Ontarians were currently in treatment for a SUD, half of whom had previous treatment history, underscoring the complex and episodic nature of SUD and the potential unmet needs of people who use substances (PWUS) [5]. Options for SUD treatment varies widely depending on the preferences and needs of the individual, the type of substances being used, the intensity or severity of their symptoms, and the availability and accessibility of services [6, 7]. SUD treatment options can include detoxification/withdrawal management, pharmacotherapy (e.g., opioid agonist treatment [OAT]), and non-pharmacological approaches such as behavior therapy (e.g., cognitive behavioral therapy [CBT]) and counselling [7, 8]. Residential programs are another treatment option, aimed at providing an integrated and holistic approach to SUD treatment that includes supporting individuals throughout the continuum of care upon treatment discharge [7, 8].

Existing literature suggests that there are two main types of residential treatment programs available across the continuum of treatment and support services: residential addiction treatment programs and residential supportive recovery programs [9]. Residential addiction treatment programs typically provide more intensive, time-limited treatment in a structured, substance-free environment [9]. Within these programs, the length of stay varies depending on client needs and the level of care provided, however, program lengths generally range from 30 to 90 days [9, 10]. Programs can include psychotherapy and clinical counselling, psychosocial education and support, and life-skills training, accompanied with 24-hour on-site access to support. While residential treatment programs have traditionally been abstinence-oriented, with documented barriers to accessing treatment for individuals on Opioid Agonist Therapy (OAT), increasingly, programs are integrating OAT into their care models, in line with current treatment guidelines that support the facilitation of evidence-based OAT alongside the ongoing provision of psychosocial supports for SUD in residential treatment settings [11, 12]. Some programs will also provide adjunct medical or clinical support in addition to OAT. Residential addiction treatment programs are typically staffed by trained clinical counsellors and program support workers, as well as nursing professionals. Many programs also have access to a physician and/or a psychiatrist.

Residential supportive recovery programs differ in that they offer substance-free accommodation accompanied by low to moderate intensity services and support, such as additional stabilization and community reintegration, and are generally considered a step down from residential addiction treatment programs [10]. Recognizing that recovery can often be a complex journey, residential supportive recovery services are a vital component in the continuum of care for SUD, as they can be accessed before or after entering more intensive treatment such as residential addiction treatment services, or by individuals who may not require clinical treatment but seek support services. They can help PWUS navigate the systems of care, and stay engaged in the recovery process by providing individuals with a safe environment to support their recovery and reintegration into the community [10]. Program lengths are generally longer than residential addiction treatment programs, and typically range from 3 to 6 months, but depends on individual needs. Supportive treatment programming typically includes peer mentoring, coaching for community reintegration, group work, education, life-skills training, and may also include basic counselling and case management [10]. These programs are commonly staffed by people with lived experience (PWLE) with substance use who have been trained to address substance use challenges. Some services may also contract external clinical counsellors or practitioners [10, 13].

However, despite the distinctions between the two residential treatment modalities, these residential treatment programs overall are not standardized and can vary greatly in terms of accessibility, type and quality of services/programming offered, program orientation/ideology, length of stays, pricing structure, and availability and provision of evidence-based interventions such as OAT. There is also limited evidence to guide providers in recommending which clients with SUD would be good candidates for residential treatment [11]. These issues can create barriers for individuals who are seeking support as it can be confusing to identify and navigate services, access information on service availability and accessibility, or even determine whether residential treatment is best suited for their needs, and suggests that the needs of PWUS are not being sufficiently met [14–18].

The availability of comprehensive, accessible information regarding the specifics of residential treatment programs is therefore crucial for individuals to make well-informed decisions about the most suitable treatment modality, level, and type. In Ontario, the primary database which houses information on residential treatment services is ConnexOntario, a health services information platform funded by the Government of Ontario that provides information on mental health, addictions, and gambling services in the province [13]. This platform provides some information regarding residential treatment programs, however additional pertinent information is not easily accessible to the general public. To obtain this information, individuals must navigate multiple online websites and contact numerous organizations to obtain this information. Considering seeking treatment is already a challenging endeavor, this may potentially deter clients and families from engaging with treatment services altogether [16]. As such, we sought to understand the scope of residential treatment programs in Ontario by conducting a provincial environmental scan of all publicly-funded residential treatment programs in the province. The goal of this scan is to provide a comprehensive overview of current residential treatment options, which can further inform policy regarding SUD treatment needs and planning in Ontario.

## Methods

### Data collection

This environmental scan was conducted with support from ConnexOntario which provided access to a back-end eService portal designed for healthcare providers to help navigate treatment options for their clients. The ConnexOntario website publicly provides information on: treatment type, services offered within programs, referral pathways, program contact information, age of eligibility, sex and/or gender eligibility, language of service provided, whether there is a cost associated with the program, and catchment area [13]. However, the back-end e-Service portal contains additional organization-level information, including specific program information, that is not publicly accessible on the ConnexOntario website. Specifically, the eService portal provides information on both assessment and enrollment wait times, program funding source, services/programming offered, program orientation/ideology, bed numbers, target demographic groups, length of stays, pricing structure, and availability and provision of evidence-based interventions such as OAT.

Between May and August 2023, we connected with ConnexOntario staff, who provided access to the ConnexOntario eService portal and an Excel list of all available publicly-funded residential addiction treatment and residential supportive recovery treatment programs in Ontario. All information on the ConnexOntario portal undergoes, at minimum, quarterly updates by respective organizations, as mandated by the Ontario government, which is further validated by healthcare data liaison personnel from ConnexOntario. While certain data, such as wait times, may receive more frequent updates, there are no specific criteria or requirements stipulating more frequent reporting for other program details.

To ensure data captured was comprehensive, we extracted all available program information. This included the following categories: organization name, geographical location, program description, program type (residential addiction treatment or residential supportive recovery), eligibility criteria, the program’s OAT policies, the number of available beds, the minimum and maximum length of stay, the current projected wait times for assessment and admission, the program’s funding source, and whether there were associated fees for program admission. Additionally, we documented the target population for each program, including whether the program was restricted for or offered accommodations for specific populations (e.g., those with hearing or visual impairments, veterans, etc.), or specialized/tailored to a particular demographic (e.g., male, female, youth, LGBTQIA+, etc.).

### Inclusion/exclusion criteria

Flexible and transitional housing programs, as well as managed alcohol programs, stabilization, or withdrawal management programs were excluded from the list. Additionally, programs entirely privately funded were excluded, except for private programs that offered publicly funded components, which were included. For example, we included specific publicly funded programs within licensed private residential treatment providers such as Bellwood Health Services and Homewood Health. Some publicly funded programs offer fee-for-service (FFS) or no-wait admission streams, and these fee-associated counterpart programs were also included.

### Data analysis and presentation

**Fig. 1:**
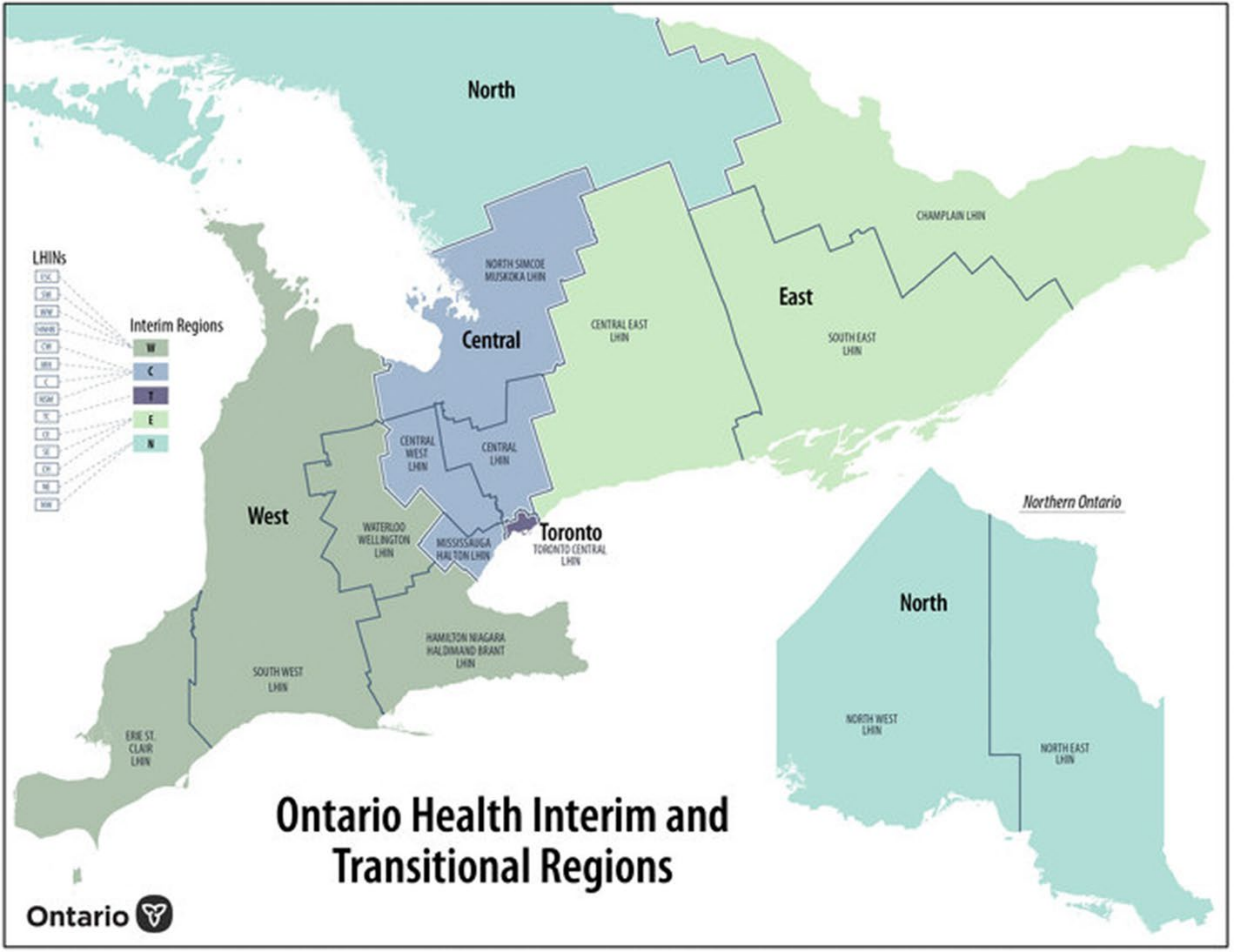
Ontario health interim and transitional regions, dissolved from the former local health integration network [19]

The data retrieved from ConnexOntario were input into an Excel document and categorized based on the type of treatment program (i.e., either residential addiction treatment or residential supportive recovery) as well as geographical location based on Ontario’s five Interim and Transitional Health regions: West, Central, Toronto, East, and North (see Fig. 1 for a regional map). In line with how ConnexOntario differentiates between programs, we categorized FFS and non-FFS counterpart programs separately to ensure comprehensive coverage of all programs offered within an organization. This approach was adopted as many organizations provide multiple treatment programs. For instance, if an organization offers both a FFS and non-FFS male residential program, they are counted and categorized as two distinct programs.

Many residential treatment programs tailor their programming to cater to specific target populations. These demographics can include members of the lesbian, gay, bisexual, transgender, queer, intersex, and asexual (LGBTQIA+) community; families, loved ones, and caregivers; veterans and members of the military; first responders; people with concurrent disorders; early childhood development; clients with legal issues; people with hearing or visual impairments; and people living with HIV. The ConnexOntario database categorizes program target populations in three primary ways: 1) programs restricted to a specific target population, 2) programs specializing in a specific target population, or 3) programs featuring components that accommodate specific target populations. There are a number of programs that overlap in the populations they specialize in and are restricted for. As an example, a program can be both restricted to and specialize in male treatment while also specializing in serving people experiencing homelessness. In such cases, males who are not experiencing homelessness may still be eligible for admission since the program is restricted to males. Program components differ from specialized programs as they offer special programming or activities to accommodate specific populations, although they may not specialize exclusively in that population.

Wait times and program lengths were recalculated in days (from weeks or months) and presented as regional averages. These wait times were further categorized into two separate categories: wait times for assessment and wait times for admission. Wait times for assessment were determined from the initial client contact, referral, or application to the program until the client was deemed eligible for admission. For instance, if a client called the service and an assessment appointment was scheduled for a later date, the wait time for the assessment would be calculated as the duration between the initial call and the assessment date. Conversely, wait times for admission were calculated from the intake appointment, where the eligibility assessment was conducted, to the commencement of the client’s program. For instance, if a 28-day program operating on a 28-day closed cycle with 10 available beds had 10 individuals on the waitlist, a newly eligible client would have to wait for two 28-day cycles to join the program, resulting in a wait time for admission of a maximum 56 days.

OAT policies were separated by methadone policies and buprenorphine policies. In the portal, each program indicated whether they offer the following methadone and buprenorphine policies (see Table 1 for summary of OAT policies captured by the portal).

**Table 1:** Summary of methadone and buprenorphine policies captured by the e-Service portal

**Methadone Policies**	
Accept Clients	The program will accept clients already taking methadone to participate in the program.
Prescribe	The program has an on-site physician who can initiate a prescription for methadone.
Dispense	Methadone is dispensed to clients via an on-site pharmacy.
Carries	The program allows methadone doses to be stored on-site.
Taper	The program has an on-site physician who manages a gradual reduction in methadone strength.
Withdrawal Management	The program will manage a client’s final stages of withdrawal from methadone.
**Buprenorphine Policies**	
Accept Clients	The program will accept clients already taking buprenorphine to participate in the program.
Prescribe	The program has an on-site physician who can initiate a prescription for buprenorphine.
Dispense	Buprenorphine is dispensed to clients via an on-site pharmacy.
Used in Withdrawal Management Protocol	Buprenorphine is used to manage a client’s rapid withdrawal from opioids.

Bed numbers, wait times, and adult program numbers were separated into three categories based on sex: male, female, and undifferentiated (any sex group). Data for bed numbers were presented both as totals by region and as rates per 100, 000 population. Some programs, particularly those offering private or FFS service options, did not allocate a specific number of beds to private-paying clients; therefore, these were counted in the total bed capacity of the program, inclusive of their public counterpart.

Most categories contained data from all programs; however, in cases where data were missing, we included the denominator to highlight the number of programs included in those specific calculations. Data were categorized and presented under the following major headings: Program target populations (including adult and youth programs, sex-specific programs, Francophone programs, programs for people experiencing homelessness, programs for family members and caregivers, and Indigenous-specific programs), wait times (including assessment and admission wait times), bed capacity, program length, substance use and OAT policies (including methadone and buprenorphine policies), program fees, and program funding.

## Findings

A total of 144 public residential treatment services were identified through the ConnexOntario eService portal. After screening, we excluded three stabilization and withdrawal management programs, two supportive and transitional housing programs, and one managed alcohol program, leaving 73 individual organizations representing 138 residential treatment services in Ontario for inclusion. These organizations offer a range of residential programs, with 102 identified as residential addiction treatment programs and 36 identified as residential supportive recovery programs.

### Regional breakdown

The West region was identified as having the largest proportion of residential treatment programs in the province at 31.4% (32/102), followed by the North at 23.5% (24/102), the East at 20.6% (21/102), Toronto at 13.7% (14/102), and the Central region at 10.8% (11/102). Regarding residential supportive recovery programs, the North region had the largest proportion at 33.3% (12/36), followed by the West region at 30.6% (11/36), the East and Toronto regions at 13.9% (5/36) each, followed by the Central region at 8.3% (3/36) .

### Program target populations

Out of 102 residential addiction treatment and 36 residential supportive recovery programs, 75.5% (77/102) and 88.9% (32/36), respectively, are restricted to specific target populations.

#### Adult and youth programs

There are 88.2% (90/102) residential addiction treatment programs designated for adults in Ontario. Of the 90 adult residential addiction treatment programs, over a third (36.7%; 33/90) of these adult programs are also accessible to clients under 18 years of age.

A small number (11.8%; 12/102) of residential addiction treatment programs are restricted to *and* specialized for youth populations, each with varying age eligibility and cut-offs. The minimum age for these programs ranges from 12 to 16, with the maximum ages extending from 17 to 25. Half (50.0%; 6/12) of these youth-specific residential addiction treatment programs are in the West region, followed by the East and Central regions (25.0%; 3/12 and 16.7%; 2/12 programs, respectively). The Toronto region does not have any youth-exclusive residential addiction treatments, however, most programs in the area accommodate individuals aged 16 and above.

In terms of residential supportive recovery programs, there are only two (5.6%; 2/36) youth-specific programs in the province, which are in the Central and West regions, respectively.

#### Sex-specific programs

Programs are either restricted to males only, females only, or offer programming to both males and females (undifferentiated). Table 2 provides a regional overview of adult residential addiction treatment programs broken down by sex in Ontario.

**Table 2:** Regional overview of adult residential treatment programs, by sex

Region	Residential Addiction Treatment (***n*** = 90)		Residential Supportive Recovery (***n*** = 34)	
	N	%	N	%
**Male only (Total)**	**33**	**36.7%**	22	64.7%
*West*	8	8.9%	7	20.6%
*Central*	6	6.7%	2	5.9%
*Toronto*	5	5.6%	5	14.7%
*East*	11	12.2%	3	8.8%
*North*	3	3.3%	5	14.7%
**Female only (Total)**	**30**	**33.3%**	9	26.5%
*West*	9	10.0%	3	8.8%
*Central*	3	3.3%	1	2.9%
*Toronto*	6	6.7%	0	0.0%
*East*	7	7.8%	2	5.8%
*North*	5	5.6%	3	8.8%
**Undifferentiated (Total)**	**27**	**30.0%**	3	8.8%
*West*	9	10.0%	0	0.0%
*Central*	0	0.0%	0	0.0%
*Toronto*	3	3.3%	0	0.0%
*East*	1	1.1%	0	0.0%
*North*	14	15.6%	3	8.8%

Programs that are inclusive of both sexes may conduct sessions with both groups together or provide specialized programming independently for each group within the program, having separate beds and programming for each. For instance, two undifferentiated residential addiction treatment programs have components specifically designed for females within the programs. One additional undifferentiated residential addiction treatment program in the North features separate sex-specific components within the program. Four additional residential addiction treatment programs offer specialized programming independently for each sex.

Two adult residential addiction treatment programs, one in the North region catering to both sexes and one in the West region restricted to males, include a component in their programming that accommodates youth. Over a third (66.7%; 8/12) of youth-specific residential addiction treatment programs accommodate both sexes; while two in the East and one in the West region are restricted to males, and one in the East is restricted to females.

Of the two youth-specific residential recovery support programs in Ontario, one is a female-only program located in the Central region, while the other program in the West region is restricted to males only.

#### Francophone programs

A very small proportion of residential addiction treatment programs, 2.9% (3/102) are restricted to French-speaking individuals (i.e., Francophones). Of these, two programs are in the North region and one program is located in the East region. Additionally, three more programs in the East offer specialized services for French-speaking individuals. One residential supportive recovery program (2.8%; 1/36) in the North region, is restricted to French-speaking individuals.

#### Programs for family members and caregivers

Several residential addiction treatment programs, 14.7% (15/102), offer components aimed at supporting families, loved ones, and caregivers of admitted clients. Further, 3.9% (4/102) of the programs, split equally between the West and Central regions, specialize in assisting families and individuals impacted by another’s substance use, while one program in the North is specifically restricted to families.

One residential supportive recovery program (2.8%; 1/36) in the North region offers a component within their program for families and other loved ones.

#### Indigenous-specific programs

Specific programs for Indigenous populations also exist, with 7.8% (8/102) of residential addiction treatment programs restricted to this group, 62.5% (5/8) of which are in the North, 12.5% (1/8) are located in the East, and 25.0% (2/8) are located in the West. Additionally, 9.8% (10/102) offer program components that accommodate for Indigenous clients, distributed across the Central (40.0%; 4/10), North (30.0%; 3/10) and West (30.0%; 3/10) regions. An additional 5.9% (6/102) of programs in the North offered specialized services for Indigenous populations.

In contrast, among residential supportive recovery programs, only one (2.8%; 1/36) program in the North is specialized for Indigenous clients, with an additional four (11.1%; 4/36) programs in the same region offering components that support Indigenous service users.

These programs typically offer a holistic treatment model that incorporates traditional and cultural activities along with Western-oriented treatment modalities. Zero residential treatment programs located in the Toronto region offer any Indigenous-specific or component programming.

### Wait times

When examining wait times, ConnexOntario categorizes this data into wait times for assessment, and wait times for admission. The average wait times for both assessment and admission across each region are outlined in Table 3.

**Table 3:** Average wait-time for assessment and admission into male, female and undifferentiated programs (in days)

Treatment Type	Region	Average Wait-Times (in Days)			
		Assessment N (Standard Deviation [SD])	Admission		
			Male Programs N (SD)	Female Programs N (SD)	Undifferentiated Programs N (SD)
**Residential Addiction Treatment (*n*** **= 96)**	**ONTARIO**	**13 ± 11.3**	**84 ± 84.0**	**74 ± 77.5**	**53 ± 39.3**
	*WEST*	12 ± 11.9	93 ± 80.3	72 ± 46.9	45 ± 17.9
	*CENTRAL*	8 ± 5.7	50 ± 41.1	62 ± 49.1	106 ± 60.1
	*TORONTO*	9 ± 9.1	69 ± 47.4	67 ± 65.4	82 ± 68.3
	*EAST*	15 ± 10.3	83 ± 82.9	75 ± 82.3	28 ± 31.8
	*NORTH*	17 ± 13.4	121 ± 154.4	90 ± 147.8	48 ± 33.4
**Residential Supportive Recovery (*n*** **= 34)**	**ONTARIO**	**10 ± 17.6**	**55 ± 59.8**	**106 ± 117.4**	**42 ± 43.7**
	*WEST*	4 ± 4.7	37 ± 29.1	91 ± 85.4	N/A
	*CENTRAL*	60 ± 42.4	52 ± 53.0	N/A	N/A
	*TORONTO*	1 ± 3.1	59 ± 61.0	N/A	N/A
	*EAST*	20 ± 10.1	175 ± 134.3	212 ± 215.0	N/A
	*NORTH*	8 ± 11.0	43 ± 32.0	51 ± 41.7	42 ± 43.7

#### Assessment wait times

Among the residential addiction treatment programs for which wait time data are available (95.1%; 97/102), the current wait times for assessment vary from 0 to 30 days, depending on the program and its capacity. More specifically, the wait times for assessment in the West, East, Central, Toronto and North regions range from 0 to 30, 0-35, 2-15, 0-30, and 1-30 days respectively.

Among the residential supportive recovery programs, data on wait times is available for 94.4% (34/36) of programs. Here, current wait times for assessment range from 0 to 90 days, depending on the program and its capacity. The range for assessment wait times in the West, East, Central, Toronto, and North regions are 0-14, 7-30, 30-90, 0-7, and 0-30 days respectively.

#### Admission wait times

Among the 96 residential addiction treatment programs with available wait time data, the current estimated wait time for admission ranged from 0 to 270 days for FFS programs and from 1 to 382 days for non-FFS programs. The current estimated wait times for admission into the 34 residential supportive recovery programs vary from 1 day to 364 days.

### Bed capacity

There are a total of 1423 residential addiction treatment beds in Ontario, translating to approximately 10 beds per 100,000 residents in the province. The North region leads in terms of residential addiction treatment bed availability, offering 34 beds per 100,000 people. It is followed by Toronto with 18 beds/100,000, the West region with 10 beds/100,000, the East region with 6 beds/100,000, and finally the Central region, which has the sparsest availability, offering 5 beds per 100,000 people. Table 4 provides an overview of the total number of beds per program type.

**Table 4:** Total number of beds by region

Treatment Type	Region	Number of Beds							
		Male		Female		Undifferentiated		Total	
		N	%	N	%	N	%	N	%
**Residential Addiction Treatment (*n*** **= 102)**	**ONTARIO**	**624**	**43.8%**	**346**	**24.3%**	**453**	**31.8%**	**1423**	**100%**
	*WEST*	164	11.5%	104	11.5%	146	10.3%	414	29.1%
	*CENTRAL*	145	10.2%	44	3.1%	63	4.4%	252	17.7%
	*TORONTO*	126	8.8%	87	6.1%	44	3.1%	257	18.1%
	*EAST*	144	10.1%	52	3.6%	18	1.3%	214	15.0%
	*NORTH*	45	3.2%	59	41.5%	182	12.8%	286	20.1%
**Residential Supportive Recovery (*n*** **= 36)**	**ONTARIO**	**361**	**76.0%**	**71**	**14.9%**	**43**	**9.1%**	**475**	**100%**
	*WEST*	86	18.1%	28	5.9%	0	0.0%	114	24%
	*CENTRAL*	50	10.5%	9	18.9%	0	0.0%	59	12.4%
	*TORONTO*	101	21.3%	0	0.0%	0	0.0%	101	21.3%
	*EAST*	39	8.2%	12	2.5%	0	0.0%	51	10.7%
	*NORTH*	85	17.9%	22	4.6%	43	9.1%	150	31.6%

Regarding residential supportive recovery beds, there are a total of 475 beds across Ontario, representing a rate of about 3 beds per 100,000 people. The North region has the highest rate with 19 beds per 100,000 people, followed by Toronto and the West region at rates of 7 and 3 beds per 100,000 people, respectively. Both the Central and the East regions have the lowest availability, each with a rate of 1 bed per 100,000 people (see Table 4).

### Program length

While the duration of some programs is flexible in length, others adhere to a fixed length of stay. Table 5 outlines a detailed view of the average minimum and maximum length of stay for both types of programs by region.

**Table 5:** Average minimum and maximum length of stay in residential treatment programs (in days)

Type of treatment	Region	Average length of stay	
		Minimum N (SD)	Maximum N (SD)
**Residential Addiction Treatment** **(*n*** **= 101)**	**ONTARIO**	**67 ± 122.9**	**100 ± 133.3**
	*WEST*	68 ± 60.1	93 ± 65.7
	*CENTRAL*	251 ± 290.9	285 ± 290.5
	*TORONTO*	39 ± 17.4	54 ± 26.8
	*EAST*	44 ± 41.9	112 ± 111.9
	*NORTH*	15 ± 21.8	37 ± 22.7
**Residential Supportive** **Recovery (*n*** **= 36)**	**ONTARIO**	**82 ± 75.3**	**286 ± 136.4**
	*WEST*	99 ± 50.9	285 ± 136.4
	*CENTRAL*	152 ± 189.8	395 ± 321.0
	*TORONTO*	108 ± 74.9	260 ± 256.0
	*EAST*	48 ± 54.0	317 ± 87.8
	*NORTH*	53 ± 49.3	256 ± 184.0

Residential addiction treatment program length greatly varies and can extend to 750 days. Programs in the North region have the shortest average minimum and maximum length of stay among all regions, averaging between 15 and 37 days. Conversely, the Central region has the lengthiest stays, with the minimum and maximum duration spanning from 251 to 285 days.

The length of stay in residential supportive recovery programs can extend to 730 days. Generally, the durations for these programs tend to exceed those of residential treatment programs in Ontario. The North region maintains the shortest average stay for residential supportive recovery programs, with durations ranging from 53 to 256 days. Conversely, the Central region exhibits the longest average stays, with durations extending from 152 to 295 days.

### Substance use policies

Nearly all programs are abstinence-based and require that clients undergo detoxification from all substances for a minimum of 72 hours prior to admission; only two residential addiction treatment programs and one residential supportive recovery program exempt clients from detoxification prior to admission.

### OAT program policies

The majority of programs (88.2%; 90/102 residential addiction treatment programs, and 97.2%; 35/36 residential supportive recovery programs) accept clients who are currently engaged in OAT. A regional overview of program OAT policies is available in Additional file 1.

Among the programs that accept clients on OAT, there is a variation in policies and procedures regarding OAT administration and maintenance. Of note, data on programs that initiate clients on OAT were unavailable. Several residential addiction treatment programs in the Toronto [2] and West [8] regions provide specialized programming to accommodate clients on OAT, which typically include on-site OAT clinics or offer specific provisions available for clients on OAT.

Among the 102 total residential addiction treatment programs, 11.8% (12/102) do not accept any clients on OAT. While all residential addiction treatment programs that allow clients on OAT (88.2%; 90/102) accept clients on buprenorphine, over a quarter 27.8% (25/90) of total programs do not accept any clients on methadone.

One residential supportive recovery program does not accept clients on OAT. Of those that do (97.2%; 35/36), over two-thirds (80.0%; 28/35) accept clients on methadone, and 97.1% (34/35) accept clients on buprenorphine.

#### Methadone policies

Among the 77 residential addiction treatment programs and the 28 residential supportive recovery programs that accept individuals receiving methadone maintenance therapy, each program has established specific policies and procedures that govern their approach. For instance, some programs (9.1%; 7/77) require clients to undergo complete detoxification and provide support during the final stages of their withdrawal process from methadone. A proportion of these programs (20.8%; 16/77 residential addiction treatment programs, and 7.1%; 2/28 residential supportive recovery programs) accept clients on methadone but have an on-site physician who manages a gradual taper for methadone. Furthermore, some residential addiction treatment programs (11.7%; 9/77) offer on-site pharmacy services that dispense methadone, while some (24.7%; 19/77) have affiliated physicians who can prescribe methadone. Lastly, some residential addiction treatment programs (35.1%; 27/77) and residential supportive recovery programs (17.8%; 5/28) permit the storage and self-administration of methadone take-home doses (THD; commonly referred to as ‘carries’) onsite. In these cases, clients take responsibility for managing their own dosing regimen. The number of programs which support each of these methadone policies is outlined in Table 6. Of the 77/90 residential addictions treatment programs and 28/35 supportive recovery programs who accept individuals on methadone, 15.6% (12/77) and 75% (21/28), respectively, did not specify what provisions or procedures are available for clients on methadone.

**Table 6:** Overview of residential treatment methadone policies among programs that accept methadone patients

		Methadone									
		Taper		Withdrawal Management		Dispense		Prescribe		Take-Home Doses	
		N	%	N	%	N	%	N	%	N	%
**Residential Addiction Treatment (*n*** **= 77)**	**ONTARIO**	**16**	**21.8%**	**7**	**9.1%**	**9**	**11.7%**	**19**	**24.7%**	**27**	**35.1%**
	*WEST*	5	6.5%	3	3.9	5	6.5	5	6.5	8	10.4%
	*CENTRAL*	0	0.0	0	0.0	0	0.0	1	1.3	2	2.6%
	*TORONTO*	9	11.7	3	3.9	3	3.9	10	13.0	3	3.9%
	*EAST*	2	2.6	0	0.0	0	0.0	2	2.6	9	11.7%
	*NORTH*	0	0.0	1	1.3	1	1.3	1	1.3	5	6.5%
**Residential Supportive Recovery (*n*** **= 28)**	**ONTARIO**	**2**	**7.1**	**0**	**0.0**	**0**	**0.0**	**0**	**0.0**	**5**	**17.8%**
	*WEST*	0	0.0	0	0.0	0	0.0	0	0.0	1	3.4%
	*CENTRAL*	0	0.0	0	0.0	0	0.0	0	0.0	1	3.4%
	*TORONTO*	2	7.1	0	0.0	0	0.0	0	0.0	2	7.1%
	*EAST*	0	0.0	0	0.0	0	0.0	0	0.0	1	3.4%
	*NORTH*	0	0.0	0	0.0	0	0.0	0	0.0	0	0.0%

#### Buprenorphine policies

Most residential addiction treatment programs (88.2%; 90/102) and residential supportive recovery programs (94.4%; 34/36) accept clients who are already on buprenorphine upon intake. This may include buprenorphine/naloxone [Suboxone] or extended-release injectable buprenorphine [Sublocade/Probuphine]). Some residential addiction treatment programs (10.0%; 9/90) offer on-site pharmacy services. Additionally, a proportion of residential addiction treatment programs (21.1%; 19/90) and one residential supportive recovery program have on-site physicians who can prescribe buprenorphine for maintenance therapy. In specific cases, some programs (6.7%; 6/90) state that they provide detoxification services to support the final stages of withdrawal from buprenorphine, while others (17.8%; 16/90) have affiliated physicians who can prescribe buprenorphine to manage rapid withdrawal from opioids. The number of programs that support each of these policies are outlined below (Table 7).

**Table 7:** Overview of residential treatment buprenorphine policies among programs that accept buprenorphine patients

		Buprenorphine							
		Used in Withdrawal Management Protocol		Withdrawal Management		Dispense		Prescribe	
		N	%	N	%	N	%	N	%
**Residential Addiction Treatment (*n*** **= 90)**	**ONTARIO**	**16**	**17.8%**	**6**	**6.7%**	**9**	**10.0%**	**19**	**21.1%**
	*WEST*	5	5.6%	2	2.2%	5	5.6%	6	6.7%
	*CENTRAL*	0	0.0%	0	0.0%	0	0.0%	0	0.0%
	*TORONTO*	7	7.8%	3	3.3%	3	3.3%	9	10.0%
	*EAST*	3	3.3%	0	0.0%	0	0.0%	2	2.2%
	*NORTH*	1	1.1%	1	1.1%	1	1.1%	2	2.2%
**Residential Supportive Recovery (*n*** **= 34)**	**ONTARIO**	**0**	**0.0%**	**0**	**0.0%**	**0**	**0.0%**	**1**	**2.9%**
	*WEST*	0	0.0%	0	0.0%	0	0.0%	0	0.0%
	*CENTRAL*	0	0.0%	0	0.0%	0	0.0%	0	0.0%
	*TORONTO*	0	0.0%	0	0.0%	0	0.0%	1	2.9%
	*EAST*	0	0.0%	0	0.0%	0	0.0%	0	0.0%
	*NORTH*	0	0.0%	0	0.0%	0	0.0%	0	0.0%

Among the 19 residential addiction treatment programs that can prescribe buprenorphine for maintenance therapy, nine (47.4%; 9/19) programs offer Sublocade, with five programs situated in the Toronto region, two in the North region, and two in the West region. Furthermore, two programs in the Toronto region also stated that they prescribe Probuphine. Of the 90 residential addictions treatment and 34 residential supportive recovery programs that accept individuals on buprenorphine, 55.6% (50/90) and 97% (33/34), respectively, did not specify what provisions or procedures are offered to clients on buprenorphine.

### Program fees

Just under one-third (28.4%; 29/102) of residential addiction treatment programs and a quarter (25.0%; 9/36) of residential supportive recovery programs are either FFS, or charge fees for various program-related costs. Within this framework, some programs reserve specific beds for clients willing to pay for immediate admission, while others impose charges for private and semi-private rooms, bed rentals, activity fees, transportation, food, and personal needs. These miscellaneous costs for services can range from $60 to $600 per month or may be determined based on the client’s income. Additionally, many programs offer bursaries to help offset program costs. FFS, private, and semi-private program costs range from $550 to $14,250 per month, with subsidies available for certain options, depending on the service.

### Program funding

Programs receive operational funding from a variety of funding sources, often combining multiple streams. The majority of these programs, specifically 68.6% (70/102) of residential addiction treatment programs and 91.7% (33/36) of supportive recovery programs primarily rely on provincial funding, predominantly sourced from Ontario Health.

For residential addiction treatment programs, additional provincial funding sources include the Ministry of Health and Long-Term Care [3], the Ministry of the Solicitor General [2], and the Ministry of Children, Community and Social Services [2]. A subset of residential treatment programs (14.7%; 15/102) receive federal funding from either Health Canada [12] or Correctional Service Canada [3]. Several residential addiction programs (22.5%; 23/102) secure funding from alternative sources such as fundraising, donations, grants, or through FFS payments.

In terms of other provincial funding sources for residential supportive recovery programs, one (2.8%; 1/36) program is funded by the Ministry of Children, Community and Social Services, while another receives funding from the Ministry of the Solicitor General. One program (2.8%; 1/36) is funded by an independent organization and one program is funded municipally by the City of Toronto. Several (22.2%; 8/36) residential supportive recovery programs rely on various other funding sources, including donations, grants, or through FFS payments.

## Discussion

This environmental scan sought to provide an overview of residential treatment programs in the province of Ontario, including key details regarding program provision and characteristics that are not publicly available, broken down by residential treatment type and geographical region. Our scan identified an unequal number of residential addiction treatment programs compared to residential supportive recovery programs across the province, with three times as many residential addiction treatment programs available compared to residential supportive recovery programs.

One of the key findings of this scan was a large variation in the geographic distribution of residential treatment programs, which underscores vast regional differences in the provision of residential treatment services. For instance, regarding residential addiction treatment programs, there is a higher concentration in the West and North compared to other regions, with 31.4 and 23.5% respectively, and these two regions also rank as the third-highest and highest, respectively, in terms of bed-per-population rates for both program types. This may indicate that compared to other regions, the availability of residential treatment programs in the West and the North is potentially more adequate and may meet the increased treatment demand occurring there. For instance, there is a higher prevalence of substance use-related morbidity and mortality in these regions, [20] and public health units within these regions have reported the largest increase in rates of opioid-related deaths between 2019 and 2020 [20]. Despite the North region boasting the most residential programs and beds, there are inconsistencies in wait times. While total wait times for supportive recovery programs in the North were among the lowest in the province, wait times for residential addiction treatment programs in that region were by far the highest. Although the higher demand for services is likely correlated with the higher prevalence of SUD in this region [18], these factors may highlight the lack of accessibility to addiction treatment services in Northern Ontario, where gaps in substance use treatment program uptake in rural and remote areas have been identified, despite the existence of these programs [14, 21]. Data suggest that rates of access to care are typically lower in rural versus urban areas due to lack of transportation and accessibility challenges, or because services tend to concentrate around larger communities [14, 22]. In this context, our data underscore the latter, where, for example, the North region covers nearly 90% of Ontario’s landmass, however, the majority of existing residential treatment programs in this catchment area are clustered within four larger urban communities: Kenora, Thunder Bay, Timmins, and Sudbury [23]. This concentration of services suggests that while there might be several residential treatment options available in these urban communities within the North, significant barriers to accessing treatment persist. These challenges encompass limited transportation support, resources and funding constraints in rural healthcare systems, as well as a shortage of qualified staff capable of providing 24-hour care in residential settings [22]. Importantly, it is worth noting that most programs serve a broad catchment area, implying that individuals across the province are generally able to access these programs. While some people may prefer the anonymity of travelling outside of their community for treatment, for others seeking treatment, this is not feasible, and could mean waiting long periods of time to attend residential treatment close to home, potentially discouraging treatment uptake. This issue may be particularly the case for individuals from rural and remote communities, where travelling outside of their immediate locality could be challenging, unfavourable, or impractical [14].

However, the regional differences in program type and availability across the province also underscore significant service gaps that correlate with extensive wait times in other areas of the province. Absolute increases in opioid deaths between 2019 and 2020 were observed in 10 other public health units, half of which are located in the East, Central, and Toronto regions [20]. The average wait times are relatively long in these three regions, especially in the East, where the average wait times for both program types are much higher than the provincial averages found in our scan. The East region also has among the lowest bed-per population rates compared to other regions. For instance, the East region had a bed-per-population rate of 1/100,000 people for residentital supportive recovery programs. Together, this may highlight increasing demands for treatment, and a growing need for programs and beds in these areas.

Differences based on sex were also found. For instance, our scan identified a substantially higher number of total available residential addiction treatment programs restricted to males only, with nearly half (44%) of the available 1423 residential treatment beds in the province specifically restricted to males, and over half (453) of the total remaining number of beds allocated to either males or females. This imbalance is also apparent when comparing addiction treatment programs to supportive recovery programs, where nearly two thirds (61%) of available residential supportive recovery services in the province are restricted to males, and there are over five times as many beds restricted to males compared to females (361 vs 71 beds, respectively). Furthermore, average admission wait times for female residential supportive recovery programs are nearly double that of male programs (106 days vs 55 days, respectively). Of importance, there are no residential supportive recovery services in the Toronto region that accept females, highlighting a critical gap in the availability of gender-inclusive addiction treatment and support options.

These sex-based variances align with existing literature, which indicates that in Ontario in 2018, a significantly larger percentage of males received substance use treatment compared to females (62% versus 38%, respectively) [5]. Moreover, in 2021, males accounted for 75% of all substance-toxicity deaths in Ontario, indicating a potentially higher demand for substance use treatment among this demographic [18]. This aligns with how treatment programs have historically accommodated males or adopted gender-neutral programming, which research has shown to benefit men more than women, highlighting the gendered nature of treatment services [24]. This may be problematic for a number of reasons, including the fact that women typically initiate substance use at a later age compared to men, and will generally experience a more rapid progression from the initiation of substance use to the development of an SUD as a result [24]. Additionally, women also present to treatment with more co-occurring psychiatric conditions, greater life instability, and greater physical and social harms associated with substance use compared to men [24]. Pregnant or parenting mothers who use substances often face additional barriers to accessing treatment, including the fear of child removal or reprisal when accessing treatment. Literature indicates that most residential programs do not allow parents to bring underage children into treatment and contact with family members and loved ones is often limited during treatment [25]. As such, emerging evidence suggests that integrating services for pregnancy and/or child-care into treatment services for women with SUD, may lead to improved parenting outcomes, such as reduced risk of maltreatment and increased parenting satisfaction [25]. In general, research has underscored the need for more gender-responsive, female-specific, SUD treatment options, and the elimination of gender-specific barriers to treatment access, such as familial responsibilities, intimate partner violence, and the heightened stigma and shame associated with accessing treatment [24, 26].

Our scan further identified a significant shortage of residential treatment programs designed for other target populations. There are only a minority of programs available for youth, each with varying age restrictions. A noteworthy observation is the absence of dedicated youth-specific programs in the Toronto region, which aligns with trends observed in the literature, where treatment programs tend to be predominantly geared towards adults [4, 27]. This may not adequately address the unique needs and vulnerabilities of young individuals who are at a heightened risk of experiencing long-term harms associated with substance use, and may require developmentally informative and age-appropriate treatment models [27]. Additionally, a very small number of programs in Ontario offer accommodation or specific programming targeted towards Indigenous clients. Indigenous people are disproportionately affected by health disparities and harms associated with substance use due to the longstanding history of colonialism, and evidence has supported the importance of integrating cultural and traditional holistic models of care in treatment services [28]. The few programs that offer such services are primarily concentrated in the North region, no doubt reflecting the larger Indigenous population living in Northern Ontario compared to the rest of the province (e.g. 16.8% Indigenous population in the North Region versus between 1.1- 4.8% in the other provincial regions) [23]. Importantly, there are zero residential services in the Toronto region that offer any Indigenous-specific accommodations or programming, despite Toronto being Canada’s largest urban centre. These findings emphasize the critical need of expanding overall treatment availability for key populations and implementing culturally sensitive and targeted programming to support diverse client profiles throughout their treatment journey [15, 16, 20, 27].

Finally, our findings shed light on immense inconsistencies in OAT policies and reveal a concerning absence of evidence-based practices within many publicly-funded residential addiction treatment programs. Specifically, when addressing treatment for opioid use, current national guidelines for OUD strongly endorse evidence-based pharmacotherapy programs such as OAT as the preferred first line of treatment across the continuum of care. It is recommended that non-pharmacological treatment approaches, including harm reduction supports and services, as well as comprehensive psychosocial treatments such as behavioural therapy, and counselling should ideally be offered in conjunction with OAT [11]. Even though most residential addiction treatment programs (88.2%) and supportive recovery programs (97.2%) accept clients who are on either methadone or buprenorphine, both of which are used in the management of OUD, most residential treatment programs did not specify whether they offered provisions to support OAT maintenance, nor was information about new initiation of OAT available. This requirement poses significant risks to clients, as relying solely on withdrawal management without linkages to long-term evidence-based care is associated with elevated risks of relapse and overdose, and it is not clinically recommended as an effective treatment for SUD [11]. Mandating withdrawal and detoxification for clients entering residential addiction treatment programs is strongly discouraged [11].

A significant area of concern lies in the considerable variations found among program-specified OAT policies, as well as the definitions of such policies. The absence of clear definitions for OAT policies poses significant challenges for clients with OUD and their families in the context of trying to navigate residential treatment programs. In the setting of an opioid overdose crisis, this lack of clarity could also result in treatment interruptions or missed opportunities for OAT initiation, both of which have been associated with increased risk of opioid overdose. These discrepancies are evident in the OAT policy definitions and the reporting system within the Connex Ontario portal, particularly in relation to the practice of conducting rapid tapers for stable clients, if OAT is being used for withdrawal management, if patient-directed tapers can be accommodated, access to OAT prescriptions, dispensed doses (commonly referred to as ‘carries’), and the specific logistics of those policies. For instance, programs differed on whether they offered on-site physicians who could prescribe or dispense carries, whether clients were allowed to store their OAT carries on-site, or if they were required to visit nearby clinics for carriers, among other distinctions. Furthermore, there are differences between the two residential treatment modalities in terms of OAT policies, with very few residential supportive recovery programs offering OAT support (i.e., only one program prescribes buprenorphine and five offer take-home doses of methadone). Although this may be due to individuals already being stabilized on OAT and thus would not require an in-house physician to prescribe, it may also reflect that individuals were required to detox and taper off OAT during residential treatment prior to residential recovery; however the available data does not elucidate these specifics. Importantly, the data on the number of programs offering OAT initiation post-admission, as well as data on OAT policies for alternative pharmacotherapies such as slow-release oral morphine (SROM), injectable OAT, and extended-release buprenorphine are also unavailable. Existing data suggest that the primary reason why residential treatments do not offer OAT initiation is due to the lack of clinical support; many programs either do not have prescribing practitioners available, or have providers who lack the necessary training and resources for OAT initiation, despite their desire for additional support and training in this regard [29, 30]. In light of the ongoing overdose crisis, marked by the increasing use of polysubstances, the importance of adhering to evidence-based practices in residential treatment cannot be overstated [29, 30]. This includes permitting, prescribing, initiating, and generally accommodating clients on OAT during residential treatment. Residential treatment programs offering OAT have been linked to improved outcomes, such as higher retention rates and reduced risk of overdose, especially among patients engaged in polysubstance use. Moreover, studies suggest that residential treatment without OAT availability results in high relapse rates with resultant increased risk for opioid-related overdose. Consequently, there is a pressing need for OAT quality standards in this sector to enhance OAT availability and accommodations within residential treatment [12, 29, 31].

Overall, our scan highlights substantial differences among residential treatment programs across the province of Ontario, underscoring a lack of standardization within programs. This variability extends to several potential barriers that could hinder access to treatment, including regional differences in availability, substantive wait times, a lack of programs tailored to specific demographics like women, youth, and other unique target populations, as well as fee variations that may impact accessibility. For instance, almost one third of residential addiction treatment programs and a quarter of residential supportive recovery programs are FFS programs or offer subsidies. This information is vital for PWUS to be able to assess affordability and explore available financial support options. Moreover, there is an overall lack of publicly-available and detailed, program-specific information. This lack of transparency poses a challenge for individuals with SUD seeking to make informed treatment decisions. As a key example, information available to the public regarding OAT policies within residential treatment programs lacks the specificity that could confirm adherence to evidence-based practices. Treatment programs must ensure that they deliver services in line with the current standard of care. Ensuring that information related to program provision is easily available, and that treatment services offered are grounded in evidence-based practice is integral in reducing barriers to accessing treatment and ensuring patient safety during treatment.

Based on the paucity of publicly available information and its potential implications for PWUS, this scan underscores the need for a comprehensive, centralized, coordinated access platform that publicly provides all essential information, complete with an integrated network of regional platforms across the province that can help streamline processes related to referrals, screening, and admissions at the local level. Such a platform would effectively minimize barriers for clients, families and/or support systems in their quest to locate, select, and access the treatments they need. As an illustrative example, *AccessMHA,* a web-based service recently developed by Regional Coordinated Access, a consortium of healthcare partners in Eastern Ontario, serves as a single-point of entry for individuals and healthcare providers within the region to connect with appropriate mental health and substance use services, simplifying the overall service navigation process [10, 32]. Although this program is in its early stages and has not been widely implemented, the Ontario Mental Health and Addictions Centre of Excellence currently has an ongoing project to scale up similar platforms across Ontario [33].

### Limitations

Several limitations of this scan should be noted. Due to the considerable variability and overlap in the definitions and categorizations of public residential treatment programs and the different levels of service delivery, it is possible that not all existing publicly-funded residential treatment programs in Ontario were captured in our scan. Additionally, our analysis was conducted based on geographic location rather than the overall program catchment areas. As such, the trends and variations noted in our analysis may be affected in part by the range of residential service categories that were included in the scan, as well as by the geographic amalgamation of the LHINS. For instance, a small number of residential addiction treatment programs included in our scan are therapeutic recovery communities, which are considered a form of long-term residential addiction treatment which also share many features of residential supportive recovery programs, such as a strong focus on peer-based support. This impacts our data on average length of stay in programs, as residential addiction treatment programs typically have much shorter length of stays. Additionally, the collapse of the North East and North West LHINs into one large “North” catchment area may have contributed to seemingly more programs in this vast area. Our analysis was further constrained by the diversity in treatment modalities, assessment criteria, and operational procedures among the programs, underscoring the lack of standardization of these programs. Consequently, we were unable to examine connections between program types, the duration of stay, services provided, and treatment intensity. Furthermore, the data collected in this scan were based on updates provided by each individual residential treatment organization/service provider in the ConnexOntario eService portal. Although these data are validated by a staff liaison at ConnexOntario, it may not have been completely up to date at the time of our analysis. While programs are mandated to update the portal quarterly, there is freedom to update the portal more frequently at program discretion, and some programs may choose to provide additional information on a more frequent basis. In addition, data on the number of programs that accommodate or specialize for other target demographic groups, such as members of LGBTQIA+ community, first responders, individuals experiencing homelessness etc., was not presented due to small sample sizes reported. Regarding OAT policies, this information was taken verbatim from the portal, and some program details were entered as multiple-choice responses to prompts provided. It is possible that the prompts have not been updated to reflect current guidelines or pharmacotherapy options, and a program may have selected information based on the only options available. For example, we noted that slow-release oral morphine (SROM) was not listed as a selection option for OAT provisions, despite being increasingly recognized and offered in treatment programs. Additional limitations around the definitions of OAT policies may exist as well, including no clear definition of the difference between taper and detox. Similarly, the portal did not offer an option to report policies surrounding OAT initiation, and we note that the language used to describe what is offered is not consistent between the buprenorphine and methadone definitions, nor is the clinical phenomenon being described clear. For example, it is not clear if rapid tapers of people previously stable on OAT are being offered, or whether OAT is being used for withdrawal management in settings where clients decline OAT. As such, the information available within the portal may not be entirely reflective of on-the-ground practices. Likewise, ConnexOntario categorizes programs by sex but not gender, which may not be reflective of the program’s practices in terms of accepting and accommodating gender-diverse populations. Lastly, the data captured on the estimated wait times do not account for factors such as individuals on the waitlist who later decline enrollment into the treatment program, as well as for other admission requirements which may not be listed in the eService portal but may affect individuals on the waitlist. For example, some programs may require the patient to call weekly for a check-in, and many vulnerable patients may be lost at this step if they happen to miss a check-in.

## Conclusion

This scan offers a comprehensive overview of Ontario’s publicly funded residential treatment landscape, shedding light on notable differences in program types and availability. Furthermore, it underscores a deficiency in readily accessible program information that is invaluable for individuals seeking residential treatment options. Residential treatment programs have the potential to significantly support individuals in their addiction journey, and providing accessible and relevant information can greatly assist PWUS in navigating the intricate and fragmented addiction treatment system. Drawing insights from the findings of this scan, we have identified several recommendations aimed at enhancing access to treatment-specific information and optimizing the provision and delivery of residential treatment services in the province.

### Supplementary Information

**Additional file 1.** Overview of program OAT policies. This table provides a regional overview of OAT policies within residential treatment programs included in this scan. This file is referenced at the beginning of page 25.

## Data Availability

All data generated or analysed during this study are included in this article [and its supplementary information files].

## Abbreviations

CBT: Cognitive Behavioural Therapy
HIV: Human Immunodeficiency Virus
LGBTQIA+: Lesbian, Gay, Bisexual, Transgender, Queer, Intersex, and Asexual
LHIN: Local Health Integration Network
OAT: Opioid Agonist Treatment
OUD: Opioid Use Disorder
PWLE: People With Lived Experience
PWUS: People Who Use Substances
SROM: Slow-Release Oral Morphine
SUD: Substance Use Disorders
THD: Take-Home Doses
