# Supplementary material for: An environmental scan of residential treatment service provision in Ontario
Source: Subst Abuse Treat Prev Policy. 2023 Dec 12;18:73. doi: 10.1186/s13011-023-00586-3 (PMC10717570; doi:10.1186/s13011-023-00586-3)
Supplement: Supplementary file 1 — Additional file 1. Overview of program OAT policies. This table provides a regional overview of OAT policies within residential treatment programs included in this scan. This file is referenced at the beginning of page 25. [file 13011_2023_586_MOESM1_ESM.docx]

Overview of program OAT policies

|  |  | **Accepts Methadone**  **Only** | | **Accepts Buprenorphine Only** | | **Accepts Both Methadone and Buprenorphine** | | **Total Accepts OAT** | | **Does Not Accept Methadone** | | **Does Not Accept Buprenorphine** | | **Total Does Not Accept OAT** | |
| --- | --- | --- | --- | --- | --- | --- | --- | --- | --- | --- | --- | --- | --- | --- | --- |
|  |  | **N** | **%** | **N** | **%** | **N** | **%** | **N** | **%** | **N** | **%** | **N** | **%** | **N** | **%** |
| **Residential Addiction Treatment (n=102)** | **ONTARIO** | **0** | **0.0%** | **13** | **12.7%** | **77** | **75.5%** | **90** | **88.2%** | **25** | **24.5%** | **12** | **11.8%** | **12** | **11.8%** |
|  | *WEST* | 0 | 0.0% | 2 | 2.0% | 23 | 22.5% | 25 | 24.5% | 9 | 8.8% | 7 | 6.9% | 7 | 6.9% |
|  | *CENTRAL* | 0 | 0.0% | 2 | 2.0% | 4 | 3.9% | 6 | 5.9% | 7 | 6.9% | 5 | 4.9% | 5 | 4.9% |
|  | *TORONTO* | 0 | 0.0% | 0 | 0.0% | 14 | 13.7% | 14 | 13.7% | 0 | 0.0% | 0 | 0.0% | 0 | 0.0% |
|  | *EAST* | 0 | 0.0% | 4 | 3.9% | 17 | 16.7% | 21 | 20.6% | 4 | 3.9% | 0 | 0.0% | 0 | 0.0% |
|  | *NORTH* | 0 | 0.0% | 5 | 4.9% | 19 | 18.6% | 24 | 23.5% | 5 | 4.9% | 0 | 0.0% | 0 | 0.0% |
| **Residential Supportive Recovery (n=36)** | **ONTARIO** | **1** | **2.8%** | **7** | **19.4%** | **27** | **75.0%** | **35** | **97.2%** | **8** | **22.2%** | **2** | **5.6%** | **1** | **2.8%** |
|  | *WEST* | 0 | 0.0% | 4 | 11.1% | 7 | 19.4% | 11 | 30.6% | 4 | 11.1% | 0 | 0.0% | 0 | 0.0% |
|  | *CENTRAL* | 1 | 2.8% | 0 | 0.0% | 2 | 5.6% | 3 | 8.3% | 0 | 0.0% | 1 | 2.8% | 0 | 0.0% |
|  | *TORONTO* | 0 | 0.0% | 1 | 2.8% | 4 | 11.1% | 4 | 11.1% | 2 | 5.6% | 1 | 2.8% | 1 | 2.8% |
|  | *EAST* | 0 | 0.0% | 2 | 5.6% | 3 | 8.3% | 5 | 13.9% | 2 | 5.6% | 0 | 0.0% | 0 | 0.0% |
|  | *NORTH* | 0 | 0.0% | 0 | 0.0% | 12 | 33.3% | 12 | 33.3% | 0 | 0.0% | 0 | 0.0% | 0 | 0.0% |
